# Spontaneous Aortocaval Fistula: A Case Report and Literature Review

**Published:** 2011-04-01

**Authors:** H Ravari, M Moini, M Vahedian, M Aliakbarian

**Affiliations:** 1 Department of Vascular Surgery, Mashhad Vascular and Endovascular Research Center, Imam Reza Hospital, Mashhad University of Medical Sciences, Mashhad, Iran; 2 Department of Vascular Surgery, Sina University Hospital, Tehran University of Medical Sciences, Tehran, Iran; 3 Department of General Surgery, Surgical Oncology Research Center, Imam Reza Hospital, Mashhad University of Medical Sciences, Mashhad, Iran

**Keywords:** Spontaneous, Aortocaval fistula, Abdominal aortic aneurysm, Iran

## Abstract

Spontaneous aortocaval fistula is a rare complication of abdominal aortic aneurysms. We describe two cases of spontaneous aortocaval fistula. The first patient is a woman who was admitted with abdominal pain and pulsatile abdominal mass. Another patient was a man admitted with progressive abdominal pain and hypotension. Computed tomography (CT) scan in both patients showed an infrarenal aortic aneurysm and simultaneous contrast enhancement in the inferior vena cava. Both patients underwent an urgent laparotomy in which the diagnosis of an aortocaval fistula was confirmed. We review the literature on spontaneous aortocaval fistula as a consequenceof complicated aortic aneurysms.

## Introduction

Spontaneous aortocaval fistula (ACF) is a pathology which occurs only in 3-6% of all ruptured abdominal aortic aneurysms. Pre-operative diagnosis is crucial. Clinical presentation is commonly acute with high output cardiac failure, chest or abdominal pain and shock.[1][2] The current report elucidates two cases of spontaneous aortocaval fistulas with the literature review.

## Case reports

### Case 1

**Fig. 1:**
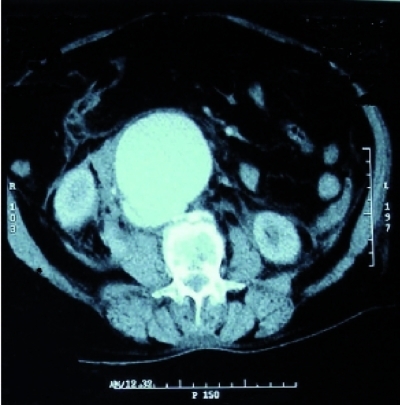
Abdominal CT scan showing infrarenal aortic aneurysm

**Fig. 2:**
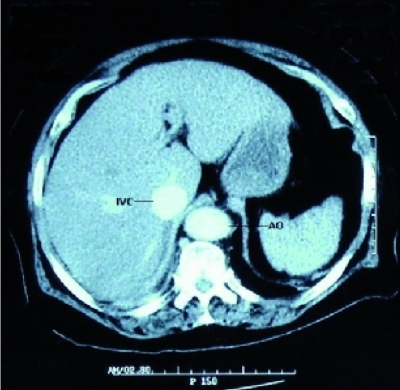
Abdominal CT scan showing simultaneous contrast enhancement in the aorta and IVC

A 67 year old female was initially presented to the Emergency Department with a complaint of epigastric abdominal pain. She described a two weeks history of that pain. Her medical history was unremarkable. On examination, she was in discomfort, with a heart rate of 90/min, blood pressure 110/60 mmHg and respiratory rate 18/min. On palpation, epigastric tenderness was present and a pulsatile mass was detected in this region. The laboratory analysis yielded hemoglobin (Hb): 11.2 g/dl (12-16), white blood cells: 14600/mm(3) (4500-11000) with neutrophils 78%. Findings on her electrocardiography and chest radiography were interpreted as normal. The patient was admitted to the Intensive Care Unit (ICU). An urgent computed tomography scan was performed which showed an infra-renal abdominal aortic aneurysm with a transverse diameter of 7 cm (Figure 1) starting 3 cm distal to renal arteries and ending 1 cm above common iliac arteries. CT findings include early detection of contrast material in the inferior vena cava, suggestive of an aortocaval fistula (Figure 2). The ACF was concealed by a huge mural thrombus.

Over the next 12 hours, the jugular venous pressure (JVP) elevated gradually, the blood pressure decreased and the patient became oliguric. Cardiac assessment did not show any pathologic change, so the patient was prepared for an emergent laparotomy. At laparotomy, a large retroperitoneal hematoma was detected and the aorta was exposed and clamped proximally from the lesser sac under the diaphragm and distally at the common iliac arteries. Having opened the aneurysm sac, the aortocaval fistula was identified. The venous bleeding into the aneurysm sac was controlled by direct pressure. The fistula was over sewn from within the aneurysm sac with 3-0 prolene. Then the aneurysm was replaced with a 20 mm diameter tubular graft. The patient was returned to the ICU after completion of the operation. She recovered very soon and was discharged in seven days.

### Case 2

A 59 year old man with a compliant of abdominal pain was referred to our department. He described a sudden onset of palpitations and dizziness, two days before admission. His past medical history was unremarkable with no history of smoking. Abdominal examination revealed a large but non-tender pulsatile mass in the epigastric region. The laboratory analysis yielded hemoglobin (Hb): 15.8 g/dl (12-16), white blood cells: 9700/mm3 (4000-11000) with 66% neutrophils, aspartate amino transferase (AST): 28 U/L (0-40), alanine amino transferase (ALT): 17 U/L (0-40) and urea: 40. After adequate resuscitation and hemodynamic stabilization, an abdominopelvic CT scan with IV contrast showed an infrarenal aortic aneurysm, 8 cm in transverse diameter, suspicious to retroperitoneal leak. It also revealed simultaneous contrast material enhancement in the inferior vena cava. Emergent laparotomy was performed and the same procedure accomplished for repair. The patient was admitted in ICU for 5 days and was discharged 7 days later.

## Discussion

There are multiple sites for rupture of abdominal aortic aneurysms in which retroperitoneum and peritoneal cavity are two common sites, although rupture into the inferior vena cava, the duodenum, the iliac vein and left renal vein may also occur. ACF is a rare pathology, which is found only in 3-6% of all ruptured abdominal aortic aneurysms.[1][2] An aortocaval fistula commonly arises from an enlarging atherosclerotic aorta, in which inflammation of the peri-aortic region results in adhesions with the adjacent inferior vena cava and subsequent pressure necrosis of the inferior wall of the vena cava. Other causes include penetrating abdominal trauma, iatrogenic trauma at lumbar disc surgery and connective tissue disorders.[3]

Because of the high pressure gradient, a sudden shift of blood occurs between high-resistance arterial and low-resistance and high-capacitance venous circuit. This results in significant decrease in the peripheral arterial resistance and an increase in the venous resistance and pressure. The myocardium hypertrophies and then dilates due to increase in the cardiac output and if untreated, it leads to irreversible hyperdynamic cardiac failure.

Reduction of arterial perfusion distal to the fistula and renal venous hypertension cause a decrease in the renal arterial perfusion pressure which in turn, activates the renin-angiotensin system. This leads to elevated secretion of aldosterone which results in plasma expansion in an attempt to increased perfusion.[4] Because of nonspecific and variable nature of the clinical symptoms, a definitive preoperative diagnosis of ACF is sometimes difficult. Clinical presentation is commonly acute but chronic complaints are also reported. Typical signs and symptoms including low back pain, palpable and pulsatile abdominal mass, abdominal bruit and thrill, dyspnea and high-output cardiac failure are present in less than 50% of cases.[5][6][7][8] Similarly, the less common ones include oliguria and consequences of regional venous hypertension (leg edema, hematuria and rectal bleeding).[9][10][11][12][13] The triad of low back pain, a palpable abdominal aortic aneurysm and a machinery abdominal murmur are diagnostic.[14][15] Diagnosis and treatment are straightforward in cases of chronic ruptures but may be difficult in cases of acute ruptures.[16]

The diagnosis in the stable patients can be confirmed in different ways. High oxygen saturations may be detected in central venous blood.[17] Doppler ultrasound will show the aortic aneurysm and may even display the fistula.[6][18] Angiography is considered the gold standard diagnostic imaging but is not appropriate in renal failure or shock.[7] Computed tomography scan, magnetic resonance imaging and radioisotope studies have all been used to make the diagnosis. Contrast CT and/or aortography may reveal early flush of contrast in the inferior vena cava from the adjacent abdominal aortic aneurysm suggesting aortocaval fistula.[18]

The prognosis of aortocaval fistula is greatly depends on early diagnosis especially before the operation. Although survival up to two months without surgery has been reported,[19] it is generally accepted that survival would be improved with prompt surgery.[17] Early diagnosis and surgery before development of shock can increase the chance of survival from 25% to 50%.[9] Diagnosis before surgery is favorable because it permits preparation of the surgeon for the appropriate surgical techniques, considering the possibility of pulmonary embolism by dislodging debris into the inferior vena cava and allows insertion of a pulmonary artery catheter for monitoring the intraoperative hemodynamic parameters[17] and preventing fluid overload which worsens cardiac failure.

Careful monitoring of perioperative hemodynamics, control of bleeding from the fistula and prevention of deep vein thrombosis and pulmonary embolism are keys to successful treatment. The location of the fistula may be determined by palpating the characteristic thrill in the inferior vena cava. Opening the aneurysm sac and evacuating the thrombus or atheroma may reveal the fistula. The most common surgical approach is the trans-peritoneal one. However, an extra-peritoneal approach through an incision in the 11th intercostal space in the left flank is another suggested option in chronic types. Surgical repair of an aortocaval fistula by endoaneurysmorraphy is now the preferred technique for fistula repair followed by prosthetic graft replacement of the aneurysm.[20] Proximal control of the aortic aneurysm should be achieved prior to distal control for avoiding a sudden increase in fistula flow. To avoid paradoxical embolization of intraluminal thrombus, minimal manipulation or mobilization of the aorta is mandatory. Venous bleeding is controlled by direct pressure (digital or swab-stick) or balloon tipped catheters (Foley or Fogarty) inserted through the fistula into the proximal and distal inferior vena cava.[4] In recent years, the endovascular approach provides a less invasive method for repair of an aortocaval fistula especially in elderly patients with several comorbidities.

In conclusion, although aortocaval fistula is a rare complication of abdominal aortic aneurysm, but its fatal nature, necessitates early diagnosis and prompt treatment. Preoperative diagnosis which is the key to successful treatment is not possible unless we keep the possibility of this complication in mind in every abdominal aortic aneurysm.
